# The effect of transcutaneous electrical stimulation of the submental area on the cardiorespiratory response in normal and awake subjects

**DOI:** 10.3389/fphys.2023.1089837

**Published:** 2023-03-14

**Authors:** Abdulaziz Alsharifi, Georgios Kaltsakas, Martino F. Pengo, Gianfranco Parati, Miquel Serna-Pascual, Gerrard Rafferty, Joerg Steier

**Affiliations:** ^1^ Centre for Human and Applied Physiological Sciences (CHAPS), Faculty of Life Sciences and Medicine, King’s College London, London, United Kingdom; ^2^ King’s College Hospital NHS Foundation Trust, London, United Kingdom; ^3^ Department of Respiratory Therapy, College of Applied Medical Sciences, Jazan University, Jazan, Saudi Arabia; ^4^ Lane Fox Unit / Sleep Disorders Centre, Guy’s and St Thomas’ NHS Foundation Trust, London, United Kingdom; ^5^ Department of Cardiology, IRCCS Istituto Auxologico Italiano, Milan, Italy; ^6^ Department of Medicine and Surgery, University of Milano-Bicocca, Milan, Italy; ^7^ Institute of Pharmaceutical Science, King’s College London, London, United Kingdom

**Keywords:** sleep apnoea, sleep-disordered breathing, upper airway physiology, hypoxia, blood pressure

## Abstract

**Background:** Electrical stimulation has recently been introduced to treat patients with Obstructive sleep apnoea There are, however, few data on the effects of transcutaneous submental electrical stimulation (TES) on the cardiovascular system. We studied the effect of TES on cardiorespiratory variables in healthy volunteers during head-down-tilt (HDT) induced baroreceptor loading.

**Method:** Cardiorespiratory parameters (blood pressure, heart rate, respiratory rate, tidal volume, airflow/minute ventilation, oxygen saturation, and end-tidal CO2/O2 concentration) were recorded seated, supine, and during head-down-tilt (50) under normoxic, hypercapnic (FiCO_2_ 5%) and poikilocapnic hypoxic (FiO_2_ 12%) conditions. Blood pressure (BP) was measured non-invasively and continuously (Finapres). Gas conditions were applied in random order. All participants were studied twice on different days, once without and once with TES.

**Results:** We studied 13 healthy subjects (age 29 (12) years, six female, body mass index (BMI) 23.23 (1.6) kg·m^−2^). A three-way ANOVA indicated that BP decreased significantly with TES (systolic: *p* = 4.93E-06, diastolic: *p* = 3.48E-09, mean: *p* = 3.88E-08). Change in gas condition (systolic: *p* = 0.0402, diastolic: *p* = 0.0033, mean: *p* = 0.0034) and different postures (systolic: 8.49E-08, diastolic: *p* = 6.91E-04, mean: *p* = 5.47E-05) similarly impacted on BP control. When tested for interaction, there were no significant associations between the three different factors electrical stimulation, gas condition, or posture, except for an effect on minute ventilation (gas condition/posture *p* = 0.0369).

**Conclusion:** Transcutaneous electrical stimulation has a substantial impact on the blood pressure. Similarly, postural changes and variations in inspired gas impact on blood pressure control. Finally, there was an interaction between posture and inspired gases that affects minute ventilation. These observations have implications on our understanding of integrated cardiorespiratory control, and may prove beneficial for patients with SDB who are assessed for treatment with electrical stimulation.

## Introduction

Obstructive sleep apnoea (OSA) is a highly prevalent condition that affects about one billion people worldwide (Benjafield et al., 2019). In patients with OSA, intermittent and repeated upper airway collapse during sleep results in irregular breathing at night. Nocturnal apnoeas and hypopnoeas lead to an altered drive to breathe, high work of breathing, oxygen desaturations, and arousals from sleep (Hilton et al., 2001). These effects can cause daytime symptoms, such as sleepiness, and are associated with increased sympathetic tone activation and elevated blood pressure (Remmers et al., 1978). OSA is associated with co-morbidities, including hypertension (Marin et al., 2005; Parati et al., 2014), ischaemic heart disease (Martinez et al., 2012), stroke (Palomäki et al., 1989), congestive heart failure (Bradley et al., 1985), obesity and metabolic syndrome (Levy et al., 2009), and diabetes (Punjabi et al., 2002).

Treatment of OSA includes continuous positive airway pressure (CPAP), and mandibular advancement devices (MAD) (National Institute for Care Excellence, 2021). Primary airway therapies aim to maintain upper airway patency during sleep and lead to a normalisation of the work of breathing and prevention of apnoeas, hypopnoeas, and arousals from sleep that could cause the sympathetic response. Long-term therapy of OSA improves daytime symptoms and, potentially, long-term cardiovascular risks (Somers et al., 2008).

CPAP therapy remains the most common treatment for moderate-to-severe OSA, while for milder cases of OSA, MADs can also be effective (NICE (National Institute for Health and Care Excellence), 2008). However, long-term adherence to CPAP therapy is limited, with only 70% adherence at 3-month (Benjafield et al., 2019b) and further reductions at later follow up (Benjafield et al., 2021). Non-CPAP therapies provide alternatives for patients who have difficulties with long-term compliance to CPAP (Randerath et al., 2021) and may be preferred over conventional treatment (Campbell et al., 2015).

Recently, electrical stimulation invasively applied using hypoglossal nerve stimulation (HNS) (Strollo et al., 2014) or transcutaneous electrical stimulation (TESLA) in the submental area to target the upper airway dilator muscles, particularly the genioglossus muscle, has been developed to treat OSA (Pengo et al., 2016). The randomised controlled trial using HNS (STAR-trial) reported modest improvements in the diastolic blood pressure with no significant changes in systolic blood pressure or heart rate over a 1-year period (Strollo et al., 2014). However, data on the acute cardiorespiratory responses to transcutaneous electrical stimulation of the upper airway dilator muscles, in both health and disease remain sparse (Pengo and Steier, 2015). This is a study to consider the physiological response to the use of electrical stimulation in direct proximity to the carotides, as hypoglossal nerve stimulation (HNS) and transcutaneous electrical stimulation is nowadays being used to treat obstructive sleep apnoea; the purpose of this study was to test the effects of the current has on the cardiorespiratory system in a cohort of normal subject (Strollo et al., 2014).

We hypothesize that acute application of transcutaneous electrical stimulation of the submental area will influence cardiovascular control in healthy, awake subjects. In the current study, we sought to describe the effect of transcutaneous electrical stimulation of the submental area on the cardiorespiratory control, for this particular purpose, we recorded beat-by-beat blood pressure with other cardiopulmonary variables when exposed to room air, hypoxic and hypercapnic gas mixtures (chemosensitivity) while in seated and supine postures, as well as with 50° HDT (baroreceptor response) while using electrical stimulation of the submental area (TES).

## Methods and subjects

The study was approved by the local research ethics committee (King’s College London; RESCM-20/21-8487) and performed in accordance with the Declaration of Helsinki. All participants received an information sheet and provided informed and written consent prior to participation.

### Subjects

We included healthy, normal- and slightly overweight subjects of both sexes over 16 years of age. All participants were non-smokers and free of cardiorespiratory and other significant acute or chronic illness and had normal blood pressure. Participants visited the respiratory physiology laboratory on two occasions at least 1 week apart, with one visit acting as control without electrical stimulation and the other during which TES was used during in all postures and gas conditions.

### Inclusion criteria

Subjects for the study met all the following criteria: age >16 years, body-mass index (BMI) > 18.5 and <30 kg/m^2^, non-smoker, and clinically stable in the last 28 days.

### Exclusion criteria

Subjects were excluded from the study if any of the following conditions were met: history of cardiovascular, respiratory, or neuromuscular disease, cardiac pacemaker, active seizures, current smokers, acute illness, allergy to skin patches, oobesity (BMI>30 kg/m^2^) or cachexia (BMI<18.5 kg/m^2^), and vertigo.

## Primary and secondary outcomes

The primary outcome of the study was the change in the diastolic blood pressure (BP) with electrical stimulation, affecting baro- and chemoreceptor response. Secondary outcomes were changes in other cardiovascular (systolic/mean BP, heart rate) and respiratory variables (respiratory rate, tidal volume, minute ventilation, modified Borg scale) during electrical stimulation.

### Equipment

**FIGURE 1:**
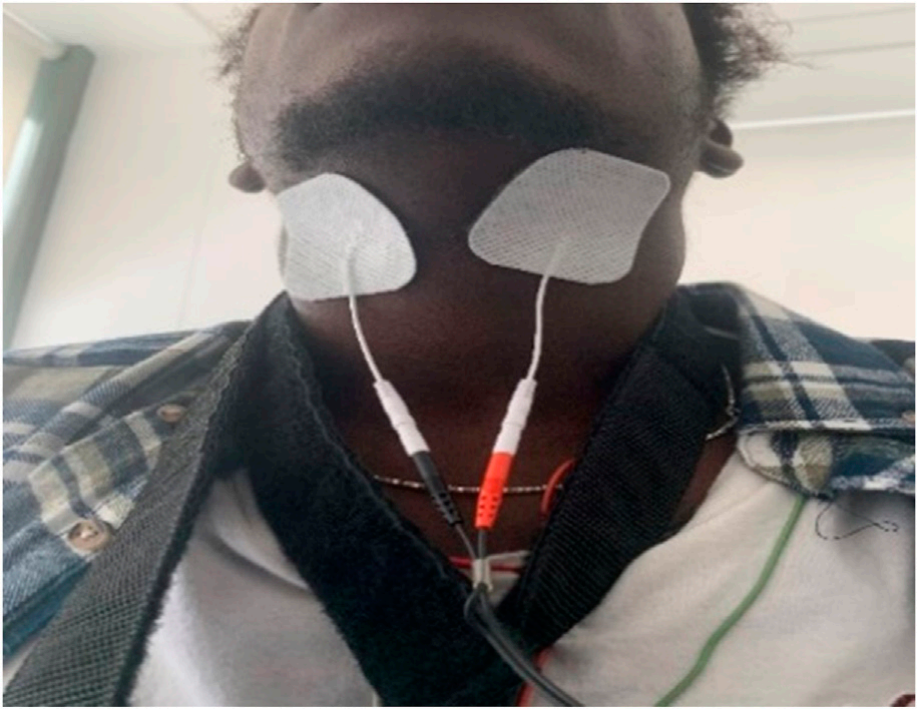
Placed in the submental area midway between angle of mandible and the chin as described previously (Steier et al., 2011).

Following the baseline visit without electrical stimulation the participants were continuously stimulated using electrical current in the submental area (4 × 4 cm dermal patches; Med-Fit Plus Ltd., Stockport, United Kingdom), at a frequency of 30 Hz and a pulse width of 250 microseconds during the second visit. Intensity of the electrical current was titrated according to individual comfort using a TENS machine (Premier Combo Plus, the TENS + Company Lets, Stockport, United Kingdom, placed in the submental area midway between angle of mandible and the chin (Figure 1) as previously described elsewhere (Steier et al., 2011). Continuous, beat-by-beat arterial blood pressure was measured continuously using digital artery photoplethysmography (Finapres, Ohmeda 2,300, BOC Healthcare, Englewood CO, United States of America). Heart rate was measured from the electrocardiogram (ECG) with electrodes positioned in the lead II configuration (ML132 bioamplifier, ADInstruments, Oxford, United Kingdom).

Respiratory flow was measured *via* a mouthpiece, with the subject wearing a noseclip using a pneumotachograph (4,800 series, Hans Rudolph Inc., Shawnee Kansas, United States of America) and associated differential pressure transducer (Spirometer, ADInstruments, Oxford, United Kingdom). The distal end of the pneumotachograph was attached to a two way non-rebreathing valve (2,700 series, Hans Rudolph Inc., Shawnee, Kansas, United States of America, deadspace 77 ml) with inspired and expired gases measured continuously using a gas analyser (ML, 206, ADInstruments, Oxford, United Kingdom), connected to a side port on the pneumotachograph *via* a fine-bore catheter. Blood oxygen saturation (SpO2) was measured using a pulse oximeter (Sat 805 pulse oximeter, Charter Kontron, United Kingdom) attached to the subject’s finger. All data were acquired (PowerLab 16, ADInstruments, Oxford, United Kingdom) with 1 Khz sampling and displayed (LabChart ver 8, ADInstruments, Oxford, United Kingdom). Tidal volume was obtained by digital integration of flow by the acquisition software.

**FIGURE 2:**
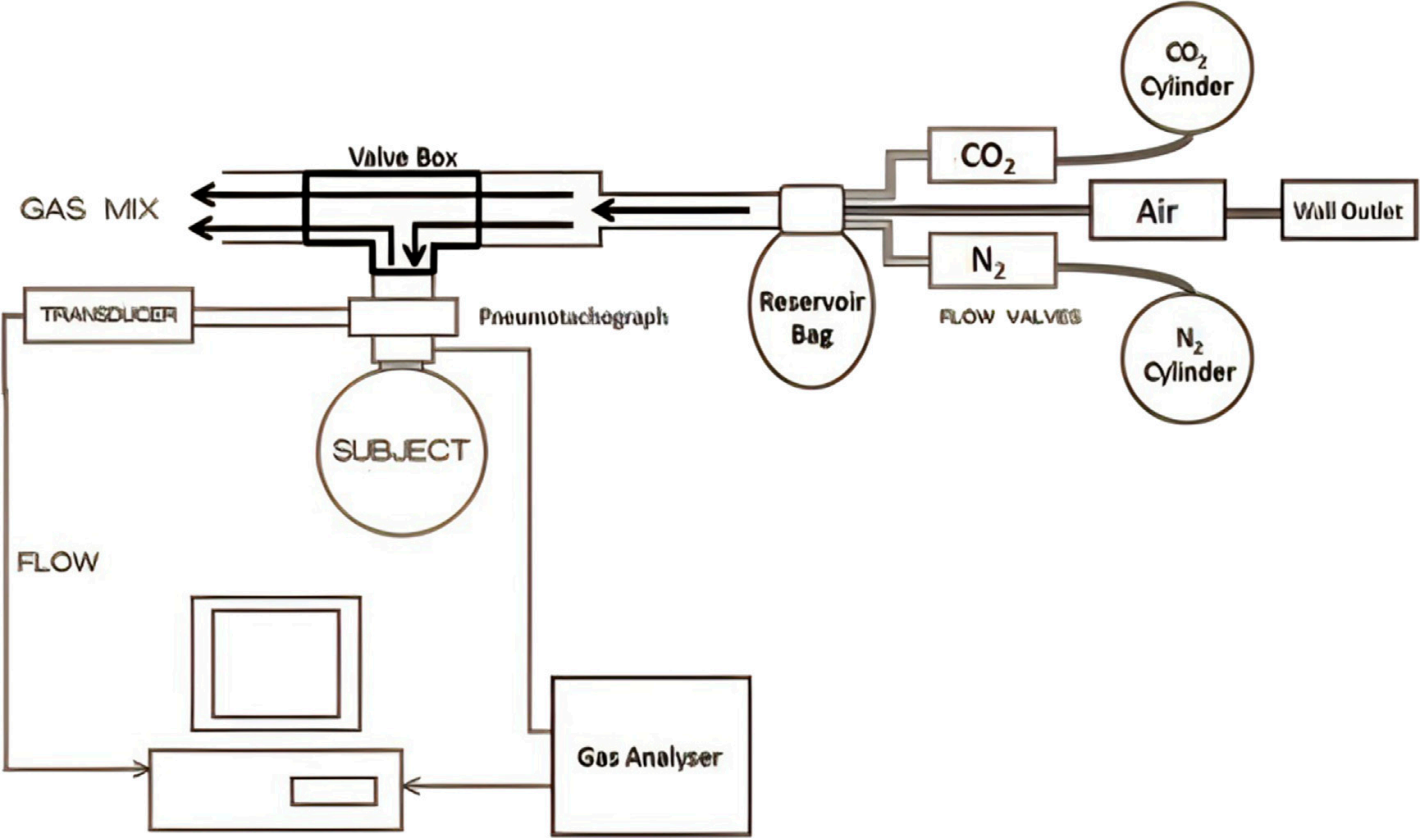
Experimental setup; and the inspiratory gas mixture could be enriched titrated (0%–100%) with 100% nitrogen or 100% carbon dioxide from cylinders (BOC, Guildford, United Kingdom).

An open circuit (Figure 2) was used to deliver a continuous supply of medical air (wall outlet) to the inspiratory port of the two-way non-rebreathing valve *via* a low volume (2.5 L) reservoir bag. The inspired gas could be enriched with 100% nitrogen or 100% carbon dioxide from cylinders (BOC, Guildford, United Kingdom) to provide the appropriate inspired gas concentration. Three inspired gas mixtures were used; medical air (21% O^2^, balance N_2_), poikilokapnic hypoxia (12% O2, balance N_2_) and normoxic hypercapnia (5% CO_2_, balance N_2_). Symptoms of breathlessness were scored using the modified Borg scale in each posture (seated, supine, and 50°HDT).

An electrically operated tilt table (Plinth2000 Ltd., Stowmarket, United Kingdom) which could be adjusted from 0° (flat) to 50°HDT was used to change posture.

## Short protocol

The following parameters were recorded at baseline: Demographic data (date of birth, height, weight, body mass index, ethnicity, and gender), clinical history, and medications. The neck, hips, and waist were measured along with vital signs (heart rate and blood pressure).

**FIGURE 3:**
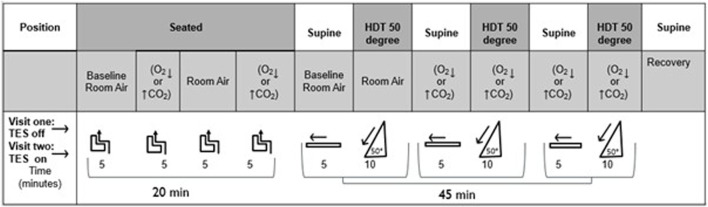
Schematic representation of study procedures. HDT: Head down Tilt. CO2: hypercapnic gas mixture (5% CO2). O2: hypoxic gas mixture (12% O2). Subjects were breathing room air, hypoxic, or hypercapnic gas mixtures in random order in seated, supine, and HDT (50) position. (off): electrical current off. (on): electrical current on (transcutaneous electrical nerve stimulation). Subjects were studied using each gas mixture for 5 min seated and supine, and for 10 min in HDT**.**

Measurements were first recorded in the seated position with the subject exposed to 5 min of each gas mixture, randomly assigned, before moving to the tilt table with measurements commencing in the supine position. Subjects were secured to the tilt table using a foam mattress and a foot strap across the ankles. Participants were familiarized with the 50°HDT procedure prior to the experiment commencing. After an initial period of stabilisation (at least 5 min) in the supine position, a period of 5 min resting breathing was recorded. The table was then tilted to the 50°HDT for 10 min. At the end of the 50°HDT, the subject was returned to the supine position for a further 5 min. Spontaneous ventilation and end-tidal gases (EtO_2_, EtCO_2_), and oxygen saturations were recorded throughout. The tilt table procedure was repeated three times with the subject breathing in random order (Figure 3). Participants were blinded to the identity of the gas being administered. For safety, the stop criterion for the hypoxic gas mixture was achieved if the arterial oxygen saturation (SpO_2_) dropped below 80%. To account for equilibration for change in posture and different gas mixtures, the final 2 min of recording for each posture and each gas mixture were analysed and an average reported for each variable.

## Data processing

All data were recorded in real-time using LabChart software (Chart V8, ADInstruments, Dunedin, New Zealand) with an analog-to-digital conversion at a sampling of 1 kHz. Data were exported and assigned key time periods for further analysis. Each variable was averaged over the last 2 min in seated, supine, and HDT positions. Respiratory variables (tidal volume (Vt) and respiratory rate (RR)) were extracted and multiplied to calculate minute ventilation (VE). Systolic (SBP), diastolic (DBP), and pulse blood pressure (pBP) was computed as pBP = SBP-DBP, and mean arterial pressure (MAP) was calculated as MAP = 1/3 SBP + 2/3 DBP. Heart Rate was derived from the 3-lead ECG, and SpO_2_ from the pulse oximeter.

### Sample size calculation

Based on the sample size of 13 subjects, the study detected a treatment difference at a two-sided significance level of 0.025 if the true mean difference in diastolic blood pressure (electrical stimulation on vs. off) was at least 8.049 mmHg (SD 10.6) with 80% power. The variable calculated was the minimal detectable difference in mean diastolic blood pressure, based on previous data (Strollo et al., 2014).

### Statistical analysis

Following testing for normality, data were presented as mean (SD) unless otherwise indicated. Data were analysed using a three-way analysis of variance (ANOVA)) followed by a Tukey’s test using the ‘anovan’ and ‘multcompare’ function of MATLAB (Version 2022B, MathWorks Ltd, Natick/MA, United States of America) to evaluate overall effects of three factors: a) TES (on/off), b) posture (seated, supine, HDT), and c) inspired gas (RA, hypercapnia, and hypoxia); furthermore, baroreflex and chemoreflex interaction was tested with the combination of these three factors. A level of significance was defined as *p* < 0.05.

## Results

We studied 13 healthy subjects (age 29 (12) years, six female, BMI 23 (1.6) kg/m^2^, waist: hip (W: H) ratio 0.87 (0.05)) (Supplementary Table S1 **)**. Two more volunteers were unable to participate in the second visit and had incomplete datasets recorded for the primary outcome, these were not included in the analysis. Subjects used an electrical current of 8 (2) mA, which had been titrated to a comfortable and tolerable level of skin sensation. There were no adverse events, and no participant required electrical stimulation to be stopped.

## Cardiovascular variables

### Systolic blood pressure

A marked reduction in systolic blood pressure during electrical stimulation was observed under hypoxic conditions in the HDT posture; there was also a trend towards reduction in other postures. (Table 1).

**TABLE 1:** Systolic blood pressure with and without electrical stimulation in seated, supine and HDT postures. Data are presented as mean (SD). HDT, head down tilt. TENS-, electrical current off. TENS + electrical current turned on. 95% CI, 95% confidence interval.

Posture	Gas mixtures	Systolic blood pressure (mmHg)			
		Visit 1 (TENS-)	Visit 2 (TENS+)	Delta Δ	95% CI
Seated	Room Air	142.33 (20.34)	127.56 (17.87)	-14.76 (22.90)	-36.10 to 6.572
	Hypercapnoea	155.44 (20.85)	139.42 (18.26)	-16.02 (23.99)	-38.37 to 6.324
	Hypoxia	148.81 (23.28)	134.69 (15.91)	-14.13 (24.52)	-36.96 to 8.713
Supine	Room Air	129.73 (20.55)	123.14 (16.62)	- 6.59 (26.05)	-30.86 to 17.68
	Hypercapnoea	137.42 (27.25)	131.12 (20.69)	- 6.29 (26.92)	-31.37 to 18.78
	Hypoxia	142.19 (20.32)	125.64 (19.01)	-16.55 (26.68)	-41.65 to 8.552
HDT 50°	Room Air	121.47 (19.69)	116.40 (21.85)	- 5.07 (23.13)	-26.62 to 16.48
	Hypercapnoea	130.61 (25.91)	116.13 (23.47)	-14.48 (27.31)	-39.92 to 10.96
	Hypoxia	132.98 (20.68)	112.03 (18.82)	-20.95 (19.97)	-39.55 to -2.349

### Diastolic and Mean Blood Pressure

A marked reduction in both diastolic and mean arterial blood pressures were also observed during electrical stimulation in supine and HDT postures. There was also a tendency towards a reduction in diastolic blood pressure during electrical stimulation when seated under hypoxic conditions, and during HDT both under hypercapnic and room air conditions. (Tables 2, 3).

**TABLE 2:** Diastolic blood pressure with and without electrical stimulation in seated, supine and HDT postures. Data are presented as mean (SD). HDT, head down tilt. TENS-, electrical current off. TENS + electrical current turned on. 95% CI, 95% confidence interval.

Posture	Gas mixtures	Diastolic blood pressure (mmHg)			
		Visit 1 (TENS-)	Visit 2 (TENS+)	Delta Δ	95% CI
Seated	Room Air	86.41 (15.89)	76.00 (13.24)	-10.41 (17.80)	-26.99 to 6.176
	Hypercapnoea	95.17 (16.55)	85.95 (11.11)	- 9.22 (14.86)	-23.07 to 4.629
	Hypoxia	90.09 (14.22)	79.50 (11.94)	-10.58 (14.71)	-24.28 to 3.122
Supine	Room Air	79.14 (11.28)	76.52 (15.20)	- 2.62 (18.22)	-19.60 to 14.35
	Hypercapnoea	88.38 (15.15)	82.23 (14.31)	- 6.15 (18.62)	-23.49 to 11.19
	Hypoxia	91.63 (11.28)	73.83 (12.20)	-17.80 (13.60)	-30.48 to -5.131
HDT 50°	Room Air	77.79 (17.32)	68.97 (20.25)	- 8.82 (20.10)	-27.54 to 9.903
	Hypercapnoea	89.14 (18.08)	71.60 (13.32)	-17.53 (19.68)	-35.87 to 0.8050
	Hypoxia	86.96 (13.69)	64.96 (12.04)	-22.01 (15.74)	-36.67 to -7.345

**TABLE 3:** Mean blood pressure with and without electrical stimulation in seated, supine and HDT postures. Data are presented as mean (SD), HDT: head down tilt. TENS-, electrical current off. TENS + electrical current turned on. 95% CI, 95% confidence interval.

Posture	Gas mixtures	Mean blood pressure (mmHg)			
		Visit 1 (TENS-)	Visit 2 (TENS+)	Delta Δ	95% CI
Seated	Room Air	101.74 (15.18)	94.67 (12.35)	- 7.07 (13.66)	-19.79 to 5.657
	Hypercapnoea	115.37 (16.92)	102.29 (13.58)	-13.08 (19.59)	-31.33 to 5.176
	Hypoxia	109.02 (17.34)	97.87 (12.33)	-11.15 (17.38)	-27.34 to 5.035
Supine	Room Air	98.42 (10.91)	91.88 (16.46)	- 4.13 (20.67)	-23.39 to 15.12
	Hypercapnoea	104.73 (18.34)	97.55 (16.59)	- 7.17 (20.78)	-26.54 to 12.19
	Hypoxia	108.49 (12.77)	89.35 (14.23)	-19.14 (15.79)	-33.85 to -4.430
HDT 50°	Room Air	92.35 (17.15)	86.01 (17.86)	- 6.34 (19.39)	-24.40 to 11.73
	Hypercapnoea	102.96 (19.13)	88.95 (11.32)	-14.01 (20.13)	-32.76 to 4.740
	Hypoxia	102.30 (14.57)	81.75 (11.82)	-20.55 (15.48)	-34.98 to -6.129

### Pulse pressure

There was no significant change to the pulse pressure when applying electrical stimulation, independent of different postures and gas mixtures. (Table 4).

**TABLE 4:** Pulse Pressure with and without electrical stimulation in seated, supine and HDT postures. Data are presented as mean (SD). HDT, head down tilt. TENS-, electrical current off. TENS + electrical current turned on. CI, 95% confidence interval.

Posture	Gas mixtures	Pulse pressure (mmHg)			
		Visit 1 (TENS-)	Visit 2 (TENS+)	Delta Δ	95% CI
Seated	Room Air	53.50 (10.11)	51.35 (13.01)	-2.15 (10.62)	-8.569 to 4.269
	Hypercapnoea	62.43 (11.75)	53.47 (13.61)	- 8.96 (10.31)	-18.58 to 0.6577
	Hypoxia	58.72 (13.31)	55.02 (11.51)	-3.69 (15.14)	-13.01 to 5.615
Supine	Room Air	49.85 (12.71)	46.69 (11.58)	-3.16 (15.64)	-11.21 to 7.208
	Hypercapnoea	48.03 (16.85)	48.88 (14.01)	0.84 (16.92)	-14.91 to 16.61
	Hypoxia	50.33 (16.14)	51.81 (15.80)	1.48 (23.18)	-12.53 to 15.49
HDT 50°	Room Air	44.01 (13.01)	47.40(16.36)	3.39(21.82)	-9.107 to 16.58
	Hypercapnoea	42.36 (19.01)	44.90(15.85)	2.53(20.84)	-16.88 to 21.96
	Hypoxia	46.01 (15.72)	47.07(16.61)	1.05(21.25)	-11.79 to 13.90

## Heart rate

The heart rate did not significantly change with electrical stimulation, and this observation was independent of posture or gas mixture. (Table 5).

**TABLE 5:** Heart rate with and without electrical stimulation in seated, supine and HDT postures. Data are presented as mean (SD). HDT, head down tilt. TENS-, electrical current off. TENS + electrical current turned on. CI, 95% confidence interval.

Posture	Gas mixtures	Heart rate (BPM)			
		Visit 1 (TENS-)	Visit 2(TENS+)	Delta Δ	95% CI
Seated	Room Air	69.65 (10.44)	73.25 (11.19)	3.60 (10.70)	-2.861 to 10.07
	Hypercapnoea	73.91 (9.90)	76.91 (12.79)	2.92 (8.01)	-7.755 to 1.909
	Hypoxia	77.71 (7.80)	79.85 (13.59)	2.14 (12.10)	-9.455 to 5.171
Supine	Room Air	62.49 (6.54)	65.38 (12.23)	2.89 (7.65)	-1.733 to 7.507
	Hypercapnoea	64.33 (7.08)	66.87 (11.71)	2.53 (9.40)	-8.216 to 3.147
	Hypoxia	72.53 (8.10)	71.56 (13.69)	- 0.97 (8.57)	-4.210 to 6.151
HDT 50°	Room Air	62.74 (10.31)	66.28 (13.15)	3.54 (7.48)	-0.9742 to 8.060
	Hypercapnoea	70.86 (17.20)	69.13 (13.78)	1.51 (12.42)	-9.023 to 12.03
	Hypoxia	74.24 (7.24)	72.51 (13.17)	-1.73 (13.17)	-3.680 to 7.151

## Respiratory variables

There was no change in the respiratory rate with electrical stimulation in any of the three postures studied. (Table 6).

**TABLE 6:** Respiratory rate with and without electrical stimulation in seated, supine and HDT postures. Data are presented as mean (SD), HDT: head down tilt. TENS-, electrical current off. TENS + electrical current turned on. CI, 95% confidence interval.

Posture	Gas mixtures	Respiratory rate (1 x min^−1^)			
		Visit 1 (TENS-)	Visit 2 (TENS+)	Delta Δ	95% CI
Seated	Room Air	17.14 (4.82)	17.24 (3.72)	0.10 (4.27)	-3.881 to 4.080
	Hypercapnoea	18.78 (4.51)	19.74 (4.65)	0.96 (3.30)	-2.114 to 4.029
	Hypoxia	18.06 (4.56)	17.07 (3.59)	- 0.99 (3.50)	-4.253 to 2.273
Supine	Room Air	17.18 (3.86)	17.25 (3.82)	0.07 (3.52)	-3.211 to 3.345
	Hypercapnoea	19.29 (3.22)	19.09 (4.28)	- 0.19 (2.62)	-2.638 to 2.252
	Hypoxia	17.83 (4.05)	15.12 (4.36)	- 2.71 (4.74)	-7.118 to 1.705
HDT 50°	Room Air	18.64 (4.12)	19.93 (4.32)	1.28 (2.66)	-1.194 to 3.762
	Hypercapnoea	20.93 (3.27)	20.18 (4.68)	- 0.75 (3.36)	-3.886 to 2.383
	Hypoxia	17.95 (4.65)	18.83 (5.83)	0.89 (5.83)	-4.373 to 6.148

### Tidal volume and minute ventilation

**FIGURE 4:**
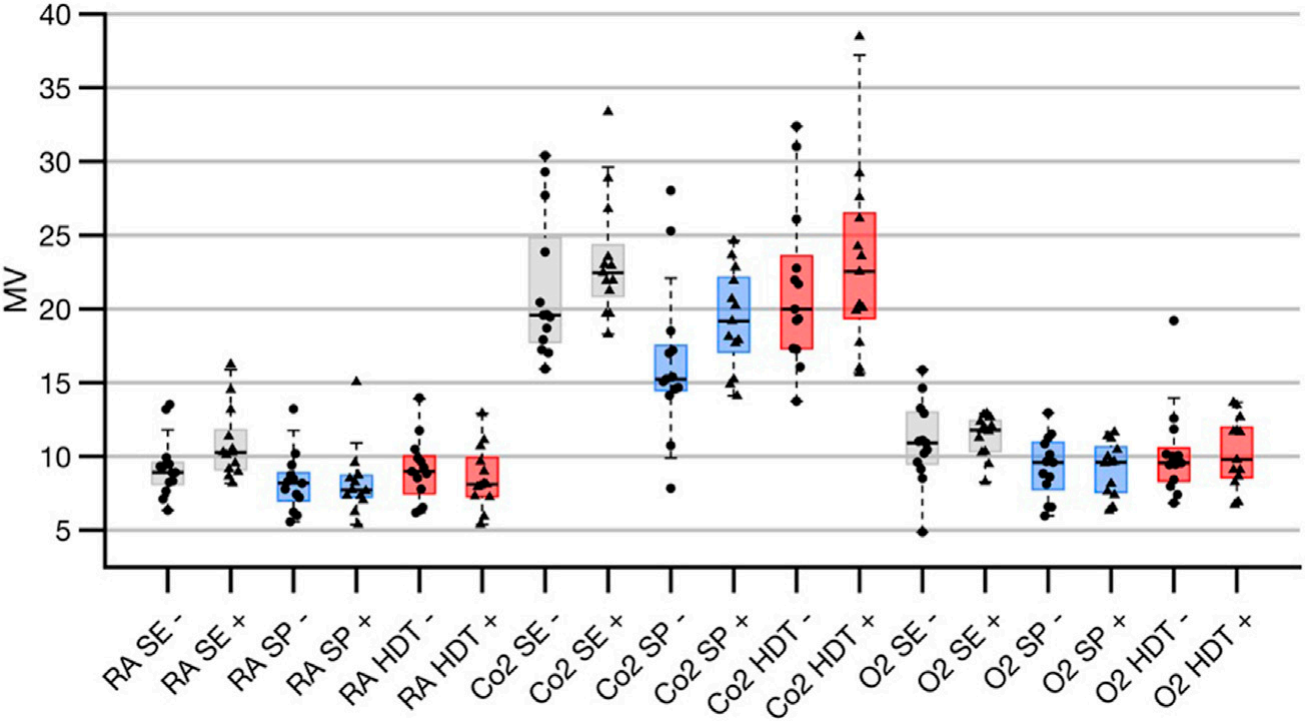
Results of 3-way ANOVA of minute ventilation derived from variation of three different factors, **(A)** TES (electrical stimulation on/off), **(B)** posture (seated, supine, HDT), and **(C)** inspired gas (room air, hypoxic and hypercapnic conditions). For further details of group assignments please refer to Supplementary Table S4. There was a significant interaction between factor b and c on minute ventilation. Data shown as median ±25 and 75% percentiles, behind individual data points.

There was a trend towards increased tidal volume in the supine posture under hypercapnic conditions with electrical stimulation, although this did not reach significance. (Table 7) Additionally, there was increased minute ventilation with electrical stimulation (p=0.0443; Table 8;Figure 4; details is provided in Supplementary Table S3).

**TABLE 7:** Tidal volume with and without electrical stimulation in seated, supine and HDT postures. Data are presented as mean (SD). HDT, head down tilt. TENS-, electrical current off. TENS + electrical current turned on. CI, 95% confidence interval.

Posture	Gas mixtures	Tidal volume (ml)			
		Visit 1 (TENS-)	Visit 2 (TENS+)	Delta Δ	95% CI
Seated	Room Air	590.47 (198.82)	684.74 (272.43)	94.27 (198.72)	- 90.86 to 279.4
	Hypercapnoea	1,217.12 (389.39)	1,239.29 (379.90)	21.39 (253.26)	- 214.5 to 257.3
	Hypoxia	653.90 (191.62)	774.35 (231.62)	120.45 (217.75)	- 82.40 to 323.3
Supine	Room Air	543.40 (251.31)	523.86 (170.34)	-19.54 (177.51)	- 184.9 to 145.8
	Hypercapnoea	903.30 (380.17)	1,107.63 (313.08)	204.33 (259.80)	- 37.69 to 446.4
	Hypoxia	587.55 (167.05)	597.86 (174.17)	10.31 (164.75)	- 143.2 to 163.8
HDT 50°	Room Air	540.18 (201.51)	468.71 (143.02)	-71.47 (190.91)	- 249.3 to 106.4
	Hypercapnoea	1,100.43 (378.97)	1,245.24 (464.19)	144.81 (381.93)	- 211.0 to 500.6
	Hypoxia	606.62 (160.60)	610.96 (151.57)	4.34 (193.44)	- 175.9 to 184.5

**TABLE 8:** Minute ventilation with and without electrical stimulation in seated, supine and HDT postures. Data are presented as mean (SD), HDT: head down tilt. TENS-, electrical current off. TENS + electrical current turned on. CI, 95% confidence interval.

Posture	Gas mixtures	Minute ventilation (L x min-1)			
		Visit 1 (TENS-)	Visit 2 (TENS+)	Delta Δ	95% CI
Seated	Room Air	9.54 (2.40)	11.17 (3.40)	1.63 (2.81)	- 0.9907 to 4.242
	Hypercapnoea	21.69 (4.55)	22.98 (3.48)	1.29 (5.36)	- 3.709 to 6.284
	Hypoxia	11.49 (3.52)	12.65 (2.59)	1.17 (4.63)	-3.149 to 5.481
Supine	Room Air	8.70 (2.54)	8.69 (2.27)	- 0.01 (2.17)	-2.028 to 2.009
	Hypercapnoea	16.86 (5.34)	20.18 (3.58)	3.33 (5.24)	-1.552 to 8.203
	Hypoxia	10.28 (3.17)	8.80 (2.74)	- 1.48 (3.62)	-4.852 to 1.897
HDT 50°	Room Air	9.55 (2.42)	9.01 (2.21)	- 0.54 (2.02)	-2.415 to 1.345
	Hypercapnoea	22.43 (5.59)	23.61 (6.16)	1.18 (4.85)	-3.340 to 5.698
	Hypoxia	10.62 (3.40)	11.15 (3.69)	0.33 (4.10)	- 3.294 to 4.351

### End-tidal carbon dioxide (ETCO_2_) and oxygen saturation

No changes in EtCO_2_ during electrical stimulation in seated, supine or HDT posture were observed when breathing room air, under hypoxic or hypercapnic conditions. The SpO_2_ did not change when comparing electrical stimulation to baseline in any posture or gas condition studied.

### Modified borg scale

There was no significant change in the breathlessness scores when applying electrical stimulation, independent of posture and gas mixture. (Supplementary Table S2).

### 3-Way ANOVA

In a 3-way ANOVA, BP decreased significantly with TES (systolic: *p* = 4.3E-06, diastolic: *p* = 3.01E-09, mean: *p* = 3.26E-08). Change in gas condition (systolic: *p* = 0.0386, diastolic: *p* = 0.0032, mean: 3.2E-03) and different postures (systolic: 6.91E-08, diastolic: *p* = 6.55E-04, mean: *p* = 4.91E-05) similarly impacted on BP control. There were no significant interactions between the three different factors: electrical stimulation, gas condition, or posture, except for an effect on minute ventilation (gas condition/posture *p* = 0.0348; Figure 4 for supplemental information on the analysis please refer to Supplementary Table S3).

## Discussion

The cardiorespiratory response to submental transcutaneous electrical stimulation, a novel therapeutic approach in OSA, applied during enhanced chemoreceptor (gas conditions) activation and baroreceptor (posture) loading demonstrates marked effects on cardiovascular control, with a modest effect on the respiratory control. Electrical stimulation appears to sensitise the arterial baroreceptor response resulting in decreased diastolic blood pressure, by 19%–25%, under hypoxic conditions (chemoreceptor) supine and with HDT (baroreceptor). The effect of electrical current on the systolic blood pressure was slightly less consistent, albeit a reduction of 16% was observed in HDT position under hypoxic conditions. There were no significant differences in the heart rate or the pulse pressure with electrical current; this was independent of posture or gas mixture used. Respiratory variables did not change significantly with electrical stimulation, except for the minute ventilation.

### Significance of findings

A number of pathways are involved in the cardiovascular responses to systemic hypoxia (Marshall, 1994), involving the primary effects of peripheral chemoreceptor stimulation, secondary effects of ventilation, and direct effects of hypoxia on the heart and peripheral vasculature leading to subsequent effects on the autonomic and the central nervous system (Marshall, 1994). The full effects of ventilation, mediated by carbon dioxide and oxygen, on the cardiovascular system remain to be fully elucidated (Heistad et al., 1974). Importantly, there is cardiorespiratory interaction which is mediated *via* hypoxia and that affects the baroreflex response, as suggested by our observations. It has been described previously that stimulation of the chemoreceptors can lead to an increased heart rate and a change in the blood pressure (cardiovagal baroreflex) in humans (Bristow et al., 1971); (Bristow et al., 1974). This is further supported by recent evidence showing that exposure to hypoxia can alter the arterial baroreflex and change heart rate and sympathetic nerve activity with a higher blood pressure. (Heistad et al., 1974); (Halliwill and Minson, 2002); (Halliwill et al., 2003).

Electrical stimulation targets the upper airway dilator muscles, particularly the genioglossus muscle (GG), and counteracts their diminished neuromuscular state-dependent tone which promotes upper airway collapsibility (Mezzanotte et al., 1996). Both the invasive and transcutaneous approaches to stimulating the upper airway dilator muscles are beneficial for maintaining airway patency during sleep in patients with OSA (Strollo et al., 2014; Pengo et al., 2016) improving the AHI by a mean of 9.1 (95% confidence interval, CI 2.0, 16.2) events/hour and the 4% ODI improved by a mean of 10.0 (95% CI 3.9, 16.0) events/hour (Pengo et al., 2016). Furthermore, the initial feasibility studies used TESLA with a current of 10.1 (3.7) mA (Steier et al., 2011). In the current study, electrical stimulation was well tolerated and had no adverse effects, underlining its safety for the use in the submental area and its efficacy in lowering diastolic blood pressure. In the context of potential long-term treatments for patients with OSA who have a high prevalence of cardiovascular comorbidities it is important to highlight that heart rate did not change significantly. A reduction in the blood pressure, systolic and diastolic, remains a favourable outcome for patients with sleep-disordered breathing, as the cardiovascular risk is typically raised and treatment resistant hypertension is of clinical relevance in this cohort (Antic et al., 2015). There are various interactions between different types of sleep apnoea and cardiovascular variables (e.g., blood pressure). On the one hand, central sleep apnoea is driven by heart failure (Javaheri and Javaheri, 2022). On the other hand, obstructive sleep apnoea leads to an increased sympathetic tone with may impact on the blood pressure contributing to hypertension (Antic et al., 2015); (Pengo et al., 2021); (Pengo et al., 2020).

### Limitations of the study

This prospective physiological study had a relatively small sample size and certain interactions could become more significant with a larger sample size, for example the effect of the three factors on minute ventilation. The effects of TES on the primary outcome variable, diastolic blood pressure, were highly consistently observed in all subjects with a large effect size. Longer steady state periods could have had further advantages over quasi-steady state achieved during the 5 min baseline periods used. The choice of this was pragmatic to allow for completion of what was a lengthy protocol and return of the healthy volunteers for a demanding second session. We were also limited with making causal inferences due to the observational nature of the design of the study. Despite a complex experimental setup, some parameters such as neural respiratory drive, blood gases, and perfusion could have provided helpful additional insights into the interaction between the cardiovascular, the respiratory, the peripheral autonomic and the central nervous system but were not measured on this occasion. In addition, this study focused on healthy subjects with normal blood pressure. Thus, further studies in subjects with hypertension and sleep-disordered breathing need to provide a comprehensive dataset on how electrical stimulation affects the chemo- and baroreceptor response in these clinically relevant cohorts. However, these points do not negate the insightful setup of a highly complex physiological experiment in human beings with a large effect size that enables to derive useful information for future clinical applications.

## Conclusion

Electrical stimulation of the submental area affects the chemo- and the baroreceptor response in normal healthy volunteers resulting in substantially lower levels of blood pressure. Similarly, inspired gas and posture impact on blood pressure regulation. Furthermore, electrical stimulation might modulate the cardiovascular risk in patients with hypertension and sleep-disordered breathing, a hypothesis that warrants further investigation in the respective clinical cohorts.

## Data Availability

The original contributions presented in the study are included in the article/Supplementary Material, further inquiries can be directed to the corresponding author.

## References

[B1] Antic N. A. Heeley E. Anderson C. S. Luo Y. Wang J. Neal B. (2015). The sleep apnea cardioVascular endpoints (SAVE) trial: Rationale, ethics, design, and progress. Sleep 38 (8), 1247–1257. 10.5665/sleep.4902 25669180 PMC4507730

[B2] Benjafield A. Oldstone L. Willes L. Kelly C. Nunez C. Malhotra A. (2021). Positive airway pressure therapy adherence with mask resupply: A propensity-matched analysis. J. Clin. Med. 10 (4), 720. 10.3390/jcm10040720 33673066 PMC7917762

[B3] Benjafield A. V. Ayas N. T. Eastwood P. R. Heinzer R. Ip M. S. M. Morrell M. J. (2019). Estimation of the global prevalence and burden of obstructive sleep apnoea: A literature-based analysis. Lancet Respir. Med. 7 (8), 687–698. [online] 7. 10.1016/s2213-2600(19)30198-5 31300334 PMC7007763

[B4] Benjafield A. V. Pepin J.-L. D. Valentine K. Cistulli P. A. Woehrle H. Nunez C. M. (2019). Compliance after switching from CPAP to bilevel for patients with non-compliant OSA: Big data analysis. BMJ Open Respir. Res. 6 (1), e000380. 10.1136/bmjresp-2018-000380 PMC6530496 31178999

[B5] Bradley T. D. Rutherford R. Grossman R. F. Lue F. Zamel N. Moldofsky H. (1985). Role of daytime hypoxemia in the pathogenesis of right heart failure in the obstructive sleep apnea syndrome. Am. Rev. Respir. Dis. 131 (6), 835–839. 10.1164/arrd.1985.131.6.835 4003933

[B6] Bristow J. Brown E. Jr Cunningham D. Goode R. Howson M. Sleight P. (1971). The effects of hypercapnia, hypoxia and ventilation on the baroreflex regulation of the pulse interval. J. physiology 216 (2), 281–302. 10.1113/jphysiol.1971.sp009525 PMC1331939 4326995

[B7] Bristow J. Brown E. Jr Cunningham D. Howson M. Lee M. Pickering T. (1974). The effects of raising alveolar PCO2 and ventilation separately and together on the sensitivity and setting of the baroreceptor cardiodepressor reflex in man. J. physiology 243 (2), 401–425. 10.1113/jphysiol.1974.sp010760 PMC1330715 4449070

[B8] Campbell T. Pengo M. F. Steier J. (2015). Patients' preference of established and emerging treatment options for obstructive sleep apnoea. J. Thorac. Dis. 7 (5), 938–942. 10.3978/j.issn.2072-1439.2015.04.53 26101652 PMC4454864

[B9] Halliwill J. R. Minson C. T. (2002). Effect of hypoxia on arterial baroreflex control of heart rate and muscle sympathetic nerve activity in humans. J. Appl. Physiology 93 (3), 857–864. 10.1152/japplphysiol.01103.2001 12183478

[B10] Halliwill J. R. Morgan B. J. Charkoudian N. (2003). Peripheral chemoreflex and baroreflex interactions in cardiovascular regulation in humans. J. physiology 552 (1), 295–302. 10.1113/jphysiol.2003.050708 PMC2343329 12897165

[B11] Heistad D. D. Abboud F. M. Mark A. L. Schmid P. G. (1974). Interaction of baroreceptor and chemoreceptor reflexes modulation of the chemoreceptor reflex by changes in baroreceptor activity. J. Clin. Investigation 53 (5), 1226–1236. 10.1172/jci107669 PMC302609 4825222

[B12] Hilton M. F. Chappell M. J. Bartlett W. A. Malhotra A. Beattie J. M. Cayton R. M. (2001). The sleep apnoea/hypopnoea syndrome depresses waking vagal tone independent of sympathetic activation. Eur. Respir. J. 17 (6), 1258–1266. 10.1183/09031936.01.00009301 11491174

[B13] Javaheri S. Javaheri S. (2022). Obstructive sleep apnea in heart failure: Current knowledge and future directions. J. Clin. Med. 11 (12), 3458. 10.3390/jcm11123458 35743529 PMC9225117

[B14] Levy P. Bonsignore M. Eckel J. (2009). Sleep, sleep-disordered breathing and metabolic consequences. Eur. Respir. J. 34 (1), 243–260. 10.1183/09031936.00166808 19567607

[B15] Marin J. M. Carrizo S. J. Vicente E. Agusti A. G. (2005). Long-term cardiovascular outcomes in men with obstructive sleep apnoea-hypopnoea with or without treatment with continuous positive airway pressure: An observational study. Lancet 365 (9464), 1046–1053. 10.1016/S0140-6736(05)71141-7 15781100

[B16] Marshall J. M. (1994). Peripheral chemoreceptors and cardiovascular regulation. Physiol. Rev. 74 (3), 543–594. 10.1152/physrev.1994.74.3.543 8036247

[B17] Martinez D. Klein C. Rahmeier L. Da Silva R. P. Fiori C. Z. Cassol C. M. (2012). Sleep apnea is a stronger predictor for coronary heart disease than traditional risk factors. Sleep Breath. 16 (3), 695–701. 10.1007/s11325-011-0559-0 21796489

[B18] Mezzanotte W. S. Tangel D. J. White D. P. (1996). Influence of sleep onset on upper-airway muscle activity in apnea patients versus normal controls. Am. J. Respir. Crit. care Med. 153 (6), 1880–1887. 10.1164/ajrccm.153.6.8665050 8665050

[B19] National Institute for Care Excellence (2021). Obstructive sleep apnoea/hypopnoea syndrome and obesity hypoventilation syndrome in over 16s [online]. https://www.nice.org.uk/guidance/ng202. 34613677

[B20] NICE (National Institute for Health and Care Excellence) (2008). Technology Appraisal Guidance (TA139). CPAP for OSA costing template and report. www.nice.org.uk/guidance/ta139.

[B21] Palomäki H. Partinen M. Juvela S. Kaste M. (1989). Snoring as a risk factor for sleep-related brain infarction. Stroke 20 (10), 1311–1315. 10.1161/01.str.20.10.1311 2799862

[B22] Parati G. Ochoa J. E. Bilo G. Mattaliano P. Salvi P. Kario K. (2014). Obstructive sleep apnea syndrome as a cause of resistant hypertension. Hypertens. Res. 37 (7), 601–613. 10.1038/hr.2014.80 24804613

[B23] Pengo M. F. Soranna D. Giontella A. Perger E. Mattaliano P. Schwarz E. I. (2020). Obstructive sleep apnoea treatment and blood pressure: Which phenotypes predict a response? A systematic review and meta-analysis. Eur. Respir. J. 55, 1901945. 10.1183/13993003.01945-2019 32079643

[B24] Pengo M. F. Steier J. Parati G. Ayas N. T. Barbé F. Barnes M. (2021). The andante project: A worldwide individual data meta-analysis of the effect of sleep apnea treatment on blood pressure. Arch. Bronconeumología 57 (11), 673–676. 10.1016/j.arbres.2021.05.002 35699002

[B25] Pengo M. F. Xiao S. Ratneswaran C. Reed K. Shah N. Chen T. (2016). Randomised sham-controlled trial of transcutaneous electrical stimulation in obstructive sleep apnoea. Thorax 71 (10), 923–931. 10.1136/thoraxjnl-2016-208691 27435610 PMC5036236

[B26] Punjabi N. M. Sorkin J. D. Katzel L. I. Goldberg A. P. Schwartz A. R. Smith P. L. (2002). Sleep-disordered breathing and insulin resistance in middle-aged and overweight men. Am. J. Respir. Crit. Care Med. 165, 677–682. 10.1164/ajrccm.165.5.2104087 11874813

[B27] Randerath W. Verbraecken J. de Raaff C. A. L. Hedner J. Herkenrath S. Hohenhorst W. (2021). European Respiratory Society guideline on non-CPAP therapies for obstructive sleep apnoea. Eur. Respir. Rev. 30 (162), 210200. 10.1183/16000617.0200-2021 34853097 PMC9489103

[B28] Remmers J. E. deGroot W. J. Sauerland E. K. Anch A. M. (1978). Pathogenesis of upper airway occlusion during sleep. J. Appl. Physiology 44 (6), 931–938. 10.1152/jappl.1978.44.6.931 670014

[B29] Somers V. K. White D. P. Amin R. Abraham W. T. Costa F. Culebras A. (2008). Sleep apnea and cardiovascular disease: An American heart association/American College of cardiology foundation scientific statement from the American heart association council for high blood pressure research professional education committee, council on clinical cardiology, stroke council, and council on cardiovascular nursing. In collaboration with the national heart, lung, and blood Institute national center on sleep disorders research (national institutes of health). Circulation 118 (10), 1080–1111. 10.1161/CIRCULATIONAHA.107.189375 18725495

[B30] Steier J. Seymour J. Rafferty G. F. Jolley C. J. Solomon E. Luo Y. (2011). Continuous transcutaneous submental electrical stimulation in obstructive sleep apnea: A feasibility study. Chest 140 (4), 998–1007. 10.1378/chest.10-2614 21454399

[B31] Pengo M. F. Steier J. (2015). Emerging technology: Electrical stimulation in obstructive sleep apnoea. J. Thorac. Dis. 7 (8), 1286–1297. 10.3978/j.issn.2072-1439.2014.04.04 26380757 PMC4561275

[B32] Strollo P. J. Jr Soose R. J. Maurer J. T. De Vries N. Cornelius J. Froymovich O. (2014). Upper-airway stimulation for obstructive sleep apnea. N. Engl. J. Med. 370 (2), 139–149. 10.1056/nejmoa1308659 24401051

