# Supplementary material for: The effect of transcutaneous electrical stimulation of the submental area on the cardiorespiratory response in normal and awake subjects
Source: Front Physiol. 2023 Mar 14;14:1089837. doi: 10.3389/fphys.2023.1089837 (PMC10043176; doi:10.3389/fphys.2023.1089837)
Supplement: Supplementary file 1 [file DataSheet1.docx]

**The effect of transcutaneous electrical stimulation of the submental area**

**on the cardiorespiratory response in normal and awake subjects**

**Results**:

We studied 13 healthy subjects (age 29 (12) years, 6 female, BMI 23 (1.6) kg/m^2^). Two more volunteers were unable to participate in the second visit and had incomplete datasets for the primary outcome, they were not included in the analysis. (**Table e-1**).

| **Subject** | **M/F** | **Age** | **weight(kg)** | **Height (m)** | **BMI (kg/m2)** | **Neck (cm)** | **Waist (cm)** | **Hip (cm)** | **W:H ratio** |
| --- | --- | --- | --- | --- | --- | --- | --- | --- | --- |
| 1 | M | 23 | 69 | 1.77 | 22.02 | 39 | 80 | 97 | 0.82 |
| 2 | F | 22 | 67 | 1.67 | 24.02 | 38 | 88 | 112 | 0.79 |
| 3 | M | 23 | 77 | 1.72 | 26.03 | 42 | 89 | 101 | 0.88 |
| 4 | F | 24 | 71 | 1.7 | 24.57 | 36 | 88 | 100 | 0.88 |
| 5 | M | 21 | 77 | 1.8 | 23.77 | 38 | 90 | 106 | 0.85 |
| 6 | F | 21 | 58 | 1.6 | 22.66 | 39 | 82 | 91 | 0.90 |
| 7 | M | 17 | 63 | 1.7 | 21.80 | 36 | 82 | 102 | 0.80 |
| 8 | M | 47 | 72 | 1.7 | 24.91 | 36 | 88 | 94 | 0.94 |
| 9 | M | 54 | 90 | 1.88 | 25.46 | 41 | 99 | 101 | 0.98 |
| 10 | F | 27 | 66 | 1.6 | 21.80 | 36 | 85 | 104 | 0.90 |
| 11 | M | 38 | 73 | 1.83 | 21.80 | 38 | 84 | 93 | 0.90 |
| 12 | F | 21 | 52 | 1.58 | 20.83 | 33 | 84 | 101 | 0.83 |
| 13 | F | 45 | 68 | 1.7 | 23.53 | 31 | 72 | 83 | 0.87 |
| **Mean** |  | 29 | 69 | 2 | 23.23 | 37 | 85 | 99 | 0.87 |
| **SD** |  | 12.14 | 9.38 | 0.09 | 1.63 | 3.00 | 6.29 | 7.36 | 0.05 |

**Table S1.** Baseline characteristics of subjects. The study cohort consisted of a mixed gender group that was young, normal in weight and slim. F: female, M: male, BMI: Body Max Index. W:H waist: hip ratio.

|  | | | Borg Scale | | | |  | |
| --- | --- | --- | --- | --- | --- | --- | --- | --- |
| Posture | **Gas mixtures** | **Visit 1 (TENS-)** | | **Visit 2 (TENS+)** | **Delta Δ** | **95% CI** | |  |
| Seated | Room Air | 0.12 (1.19) | | 0.08 (0.19) | -0.038 (0.14) | -0.1677 to 0.0907 | |  |
|  | Hypercapnoea | 1.38 (1.36) | | 2.19 (0.99) | 0.808(1.16) | -0.2770 to 1.892 | |  |
|  | Hypoxia | 0.62 (0.85) | | 0.62 (0.71) | 0.000(0.37) | -0.3294 to 0.3294 | |  |
| Supine | Room Air | 0.15 (0.38) | | 0.15 (0.38) | 0.076(0.28) | -0.1815 to 0.3353 | |  |
|  | Hypercapnoea | 1.27 (1.36) | | 1.27 (1.11) | 0.000 (1.00) | -0.8919 to 0.8919 | |  |
|  | Hypoxia | 0.35 (0.55) | | 0.42 (0.57) | 0.077 (0.36) | -0.2439 to 0.3977 | |  |
| HDT  50° | Room Air | 0.31 (0.63) | | 0.65 (0.83) | -0.346(1.13) | -1.359 to 0.6670 | |  |
|  | Hypercapnoea | 2.15 (1.33) | | 2.00 (0.89) | -0.154(1.40) | -1.421 to 1.113 | |  |
|  | Hypoxia | 0.81 (0.78) | | 0.92 (1.00) | 0.115 (1.09) | -0.8755 to 1.106 | |  |

**Table S2.** Modified Borg scale with and without electrical stimulation in seated, supine and HDT postures. Data are presented as mean (SD). HDT, head down tilt. TENS-, electrical current off. TENS+ electrical current turned on. CI, 95% confidence interval.

**Table S3**. 3-way ANOVA and post-hoc Tukey’s tests MATLAB output. Stimulation: (TES ON *VS* OFF). Condition: inspired gas (Room air (1), hypercapnia (2), and hypoxia (3)). Position: (Seated (1), Supine (2), Head down tilt (3)). Sum Sq. (The sum of squares due to each source). d.f (Degree of freedom associated with each source). Mean Sq, the mean squares for each source, which is the ration SS/df. Prob F: the p-value.

**Table S4**. Studied groups numeration. (no): Electrical stimulation off. (yes): Electrical stimulation on. (HDT): Head down tilt. (RA): Room air. (CO2): hypercapnic. (O2): hypoxic.
